# Influence of Pump Current Waveform on The Mitigation of Transverse Mode Instability in Fiber Laser Oscillator

**DOI:** 10.3390/mi14040864

**Published:** 2023-04-17

**Authors:** Junyu Chai, Wenguang Liu, Xiaolin Wang, Qiong Zhou, Jiangbin Zhang, Hanwei Zhang, Pengfei Liu, Yao Lu, Dan Zhang, Zongfu Jiang, Guomin Zhao

**Affiliations:** 1 College of Advanced Interdisciplinary Studies, National University of Defense Technology, Changsha 410073, China; junyuchai@nudt.edu.cn (J.C.); lwg.kevin@163.com (W.L.); zhangjiangbin@nudt.edu.cn (J.Z.); zhanghanwei100@163.com (H.Z.); jiangzongfu7@163.com (Z.J.);; 2 Nanhu Laser Laboratory, National University of Defense Technology, Changsha 410073, China; 3 Hunan Provincial Key Laboratory of High Energy Laser Technology, Changsha 410073, China

**Keywords:** transverse mode instability, pump modulation, current waveform, fiber laser oscillator

## Abstract

We carry out a detailed investigation of TMI mitigation by pump modulation based on multiple current waveforms in a fiber laser oscillator. Compared with continuous wave (CW), the modulation of various waveforms, including sinusoidal wave, triangular wave, and pulse wave with a duty cycle of 50% and 60%, can increase the TMI threshold. The average output power of a stabilized beam is boosted via the adjustment of phase difference between the signal channels. The TMI threshold is increased to 270 W under a modulation of pulse wave (duty cycle: 60%) with a phase difference of 440 μs, where the beam quality is 1.45. This threshold can be further improved by adding groups of pump LDs and drivers, which is a promising approach for beam stabilization of high-power fiber lasers.

## 1. Introduction

Fiber lasers have become widely used in medicine, industry, and science due to their good performance. This great demand has led to an unparalleled exponential evolution of the average and peak power of fiber laser systems over the past two decades [1,2,3,4]. However, these impressive developments have caused a drastic increase in the thermal load on such systems, inducing thermal effects in the active fibers [5,6]. Under the influence of thermal effects, there are two possible changes in a fiber laser system. One is progressive shrinking of the mode size, i.e., the mode field diameter, with increasing average power [7]. This intensifies the impact of nonlinear effects, cutting down the benefit of a large fiber core [8]. The other is the occurrence of transverse mode instability (TMI), which is a manifestation of thermally-induced nonlinear effects [9,10,11,12]. TMI has been demonstrated to be the strongest limitation on the average power scaling of fiber laser systems. It leads to a sudden deterioration of beam quality and stability when reaching a certain power threshold [13,14,15]. The beam fluctuations are dynamic energy transfers between different orthogonal transverse modes in an optical fiber [16,17]. It is widely accepted there are two compulsory requirements to fulfill the energy transfer, namely, the appearance of a refractive index grating (RIG) generated by a modal interference pattern (MIP), and a subsequent phase shift between the RIG and MIP [18,19,20]. The sign of the phase shift (the position of the maximum of MIP relative to the maximum of RIG) determines the direction of the modal energy transfer [21,22,23,24]. A positive (negative) phase shift causes an energy transfer from higher-order modes (fundamental mode) to fundamental mode (higher-order modes). If the maximum and minimum of the MIP and RIG are aligned, i.e., there is no shift, then no energy exchange between the fiber modes can occur. This phase shift can be controlled by heat load modulation through the modification of pump power [25,26]. Thus, if the pump power is properly modulated, TMI can be suppressed.

There have been several studies of TMI mitigation strategies based on pump modulation [8,21,22,23,24,25,26,27]. They have mainly focused on the influence of pump power and modulation frequency on the TMI threshold. However, the mitigation effect of various current waveforms based on the modulation of multiple parameters has not been researched and compared clearly. In order to fill this gap, in this study we carry out a number of experiments involving adjusting the waveform of the pump current. We find that the modification of pump current waveforms can increase the TMI threshold. The average output power of a stabilized beam can be further boosted via the phase difference between the two channels of the function generator.

## 2. Experimental Setup

**Figure 1:**
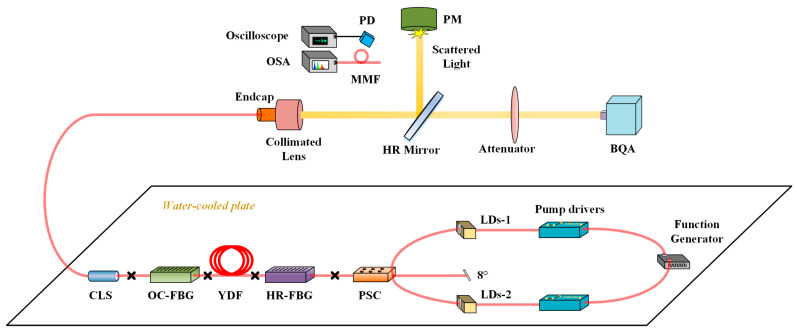
Experimental setup containing fiber laser oscillator and pump modulation devices. (LDs: Laser diodes; PSC: Pump-signal combiner; HR: High reflection; FBG: Fiber Bragg grating; OC: Output coupler; YDF: Ytterbium-doped fiber; CLS: Cladding light stripper; PD: Photodiode; OSA: Optical spectrum analyzer; MMF: Multimode fiber; PM: Power meter; BQA: Beam quality analyzer).

As depicted in Figure 1, we designed an experimental setup which contains a co-pumped fiber laser oscillator and pump modulation devices. The pump modulation devices include a dual-channel function generator and two pump drivers.

The pump sources are multiple fiber-coupled LDs with a stabilized emission wavelength of 976 nm. Two groups of pump LDs are merged into the laser cavity through the forward pump-signal combiner (PSC). The central pump port of the PSC is angle cleaved to avoid the return light. There are a high-reflection fiber Bragg grating (HR-FBG) and an output coupler fiber Bragg grating (OC-FBG) inscribing on the two ends of Ytterbium (Yb)-doped fiber (YDF). The HR-FBG provides a reflectivity of ~99.9% with a 3 dB bandwidth of ~4 nm at the center wavelength of ~1080 nm, while the OC-FBG provides a reflectivity of ~10% with a 3 dB bandwidth of ~1.7 nm at the center wavelength of ~1080 nm. The length of the YDF (core/cladding diameter: 30/400 μm, NA_core_: 0.064) is ~20 m. It is coiled in the shape of a figure-eight with a minimum diameter of 85 mm. A length of 3 m of delivery fiber (core/cladding diameter: 30/400 μm, NA_core_: 0.064) is spliced after the OC-FBG. There is a cladding light stripper on the delivery fiber to filter the cladding light. The entire experimental setup is placed on a water-cooled plate. The laser is emitted from a quartz endcap and is characterized using a photodiode (PD) and oscilloscope, optical spectrum analyzer (OSA), and beam quality analyzer (BQA).

## 3. Results and Discussion

**Figure 2:**
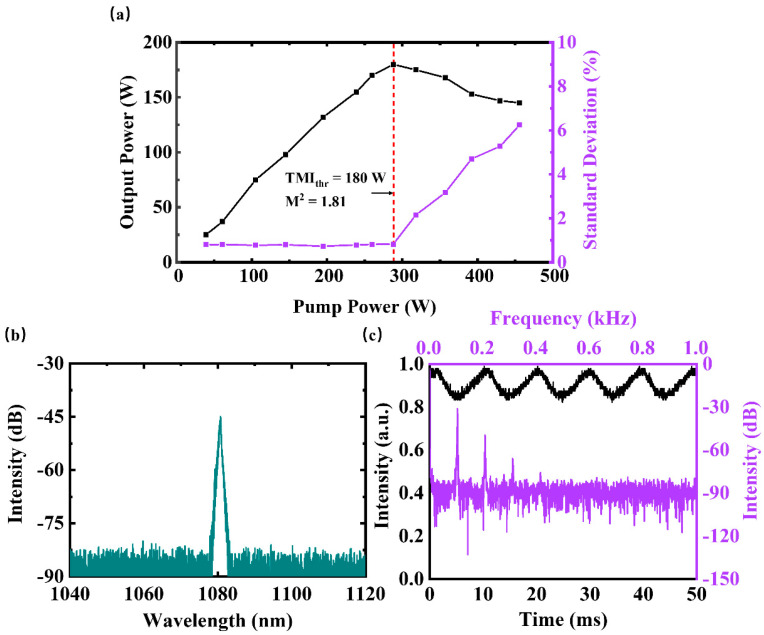
The output characteristics of the deployed fiber laser oscillator: (**a**) the curves of the output power (black line) and standard deviation versus the pump power (purple line); (**b**) the spectrum at 180W (blackish green line); (**c**) temporal traces (black line) and the related Fourier spectrum (purple line) at the onset of TMI.

We first measure the output characteristics of the fiber laser oscillator in CW mode. Figure 2a shows that the TMI threshold is ~180 W, as the standard deviation of temporal signals sharply increases after this rollover output power. Figure 2b,c depicts the spectrum of the output laser at 180 W and the temporal traces at the onset of TMI. The central wavelength is around 1080 nm, and there is no stimulated Raman scattering (SRS) or amplified spontaneous emission (ASE). The fluctuation of the temporal signal approximates the sinusoidal wave. According to the related Fourier spectrum, the main characteristic frequency is about 100 Hz, and there are several other individual characteristic peaks below 500 Hz.

**Figure 3:**
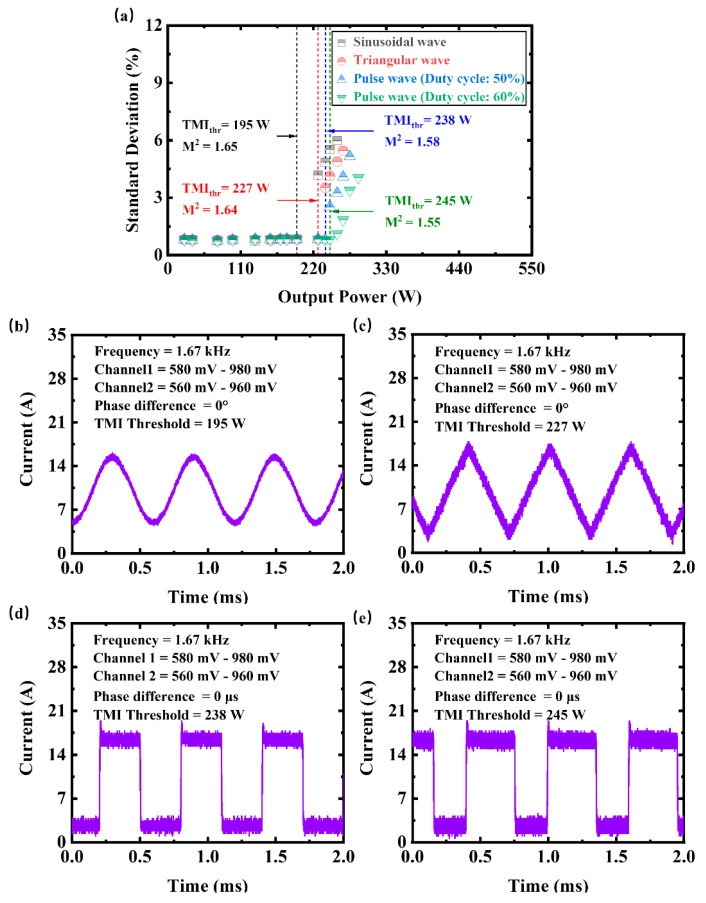
The output characteristic under the pump modulation of various waveforms with no phase difference between two channels of the function generator. (**a**) The evolution of the standard deviation of the beam fluctuations with the output power (the modulation frequency and its harmonics for the raw signals have been filtered out to calculate the temporal stability). Black dot: sinusoidal wave modulation. Red dot: triangular wave modulation. Blue dot: pulse wave (duty cycle: 50%) modulation. Green dot: pulse wave (duty cycle: 60%) modulation. The total current (purple line) of pump LDs at TMI threshold under the modulation of (**b**) sinusoidal wave, (**c**) triangular wave, (**d**) pulse wave (duty cycle: 50%), and (**e**) pulse wave (duty cycle: 60%).

To research the influence of the pump current waveform on the TMI threshold, each channel of the function generator was plugged into a pump driver with a group of LDs, with the modulation frequency picked according to the frequency of the phase change. According to the [28], the frequency of phase change is related to the thermal response time of an optical fiber. The thermal response time can be expressed as $\frac{d^{2}}{D}$, where *d* is the core diameter and *D* denotes the thermal diffusion coefficient. For silica optical fibers, the thermal diffusion coefficient is $D=8.46\times 10^{-7}m^{2}/s$. Thus, theoretically, the modulation frequency to stabilize the beam of our system needs to be around 1 kHz. Based on repeated experiments, the appropriate frequency is.1.67 kHz. The waveform of the output laser relies on the pump current. The beam fluctuations induced by TMI are amplitude noise in the output laser. This amplitude noise needs to be extracted from the raw signals by filtering the main frequency and harmonics, as the input signals are not perfect waveforms. The standard deviation of the amplitude noise is calculated to analyze the temporal stability of the beam. Figure 3a shows the evolution of the standard deviation of the beam fluctuations, while Figure 3b depicts the total current of pump LDs at the TMI threshold under the modulation of various waveforms with no phase difference between two signal channels. The amplitude range of signals to reach a higher TMI threshold is 580–980 mV for channel 1 of the function generator and 560–960 mV for channel 2. When the same signal amplitude is applied after converting the current waveform, the total response current of LDs has certain divergences (sinusoidal wave: 4.3–16.1 A; triangular wave: 3.7–17.1 A; pulse wave (duty cycle: 50%): 1–19.1 A; pulse wave (duty cycle: 60%): 0.7–19.8 A). This response difference is due to the current noise of each waveform. When the input signal is pulse wave (duty cycle: 60%) with a current range of 0.7–19.8 A, the maximum average power of a stabilized beam can reach 245 W, which is about 1.3 times the TMI threshold in CW mode. The beam quality is about 1.55.

**Figure 4:**
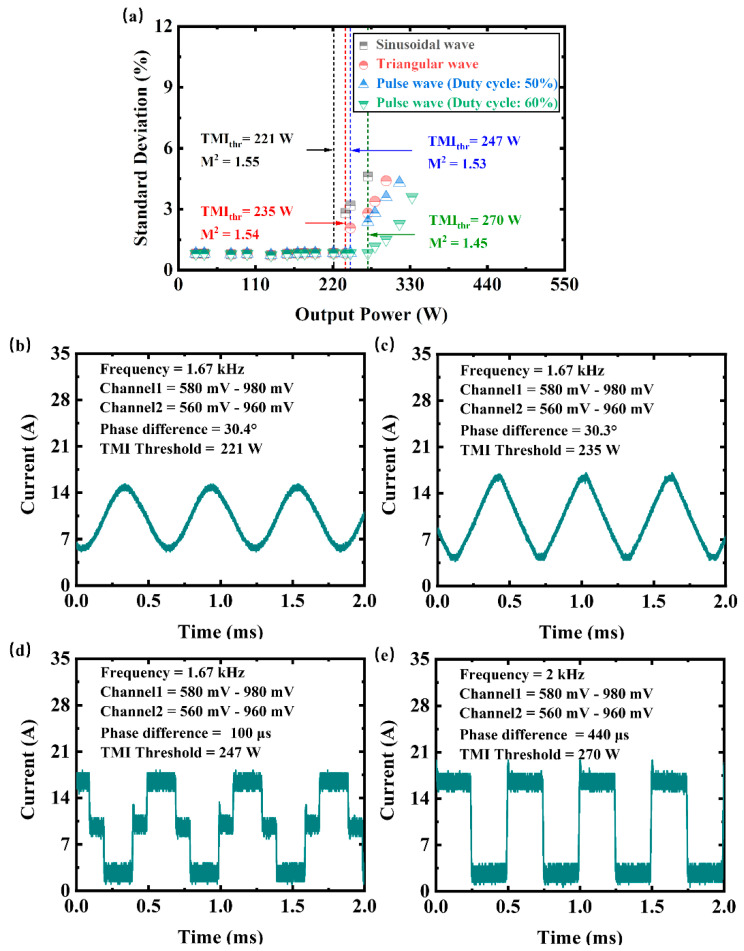
The output characteristic under the pump modulation of various waveforms with a certain phase difference between two channels of the function generator. (**a**) The evolution of the standard deviation of the beam fluctuations with the output power (the modulation frequency and its harmonics for the raw signals have been filtered out to calculate the temporal stability). Black dot: sinusoidal wave modulation. Red dot: triangular wave modulation. Blue dot: pulse wave (duty cycle: 50%) modulation. Green dot: pulse wave (duty cycle: 60%) modulation. The total current (blackish green line) of pump LDs at TMI threshold under the modulation of (**b**) sinusoidal wave, (**c**) triangular wave, (**d**) pulse wave (duty cycle: 50%), and (**e**) pulse wave (duty cycle: 60%).

Furthermore, we record the change of output characteristics by adjusting the phase difference, as depicted in Figure 4. Here, the phase difference is the lead or lag in time between the signals emitted by two channels of a function generator. The TMI threshold is further increased with adjustment of the phase difference under the same signal amplitude for each current waveform. In addition, multiple modulation frequencies can maximize the output power, e.g., 2 kHz is another optimal frequency for the modulation of pulse wave (duty cycle: 60%), as shown in Figure 4e. In this case, the maximum output power of the stabilized beam can reach 270 W under a phase difference of 440 μs, which is about 1.5 times the TMI threshold in CW mode. The beam quality is about 1.45.

**Figure 5:**
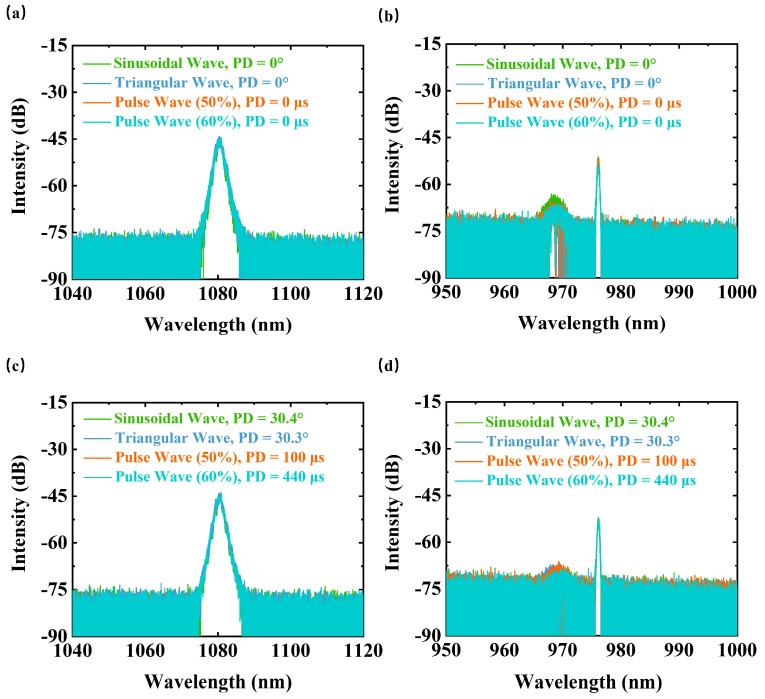
The laser output spectrum (**a**) and pump laser spectrum (**b**) based on pump modulation of various waveforms without phase difference. The laser output spectrum (**c**) and pump laser spectrum (**d**) based on pump modulation of various waveforms with a certain phase difference. PD: phase difference.

The laser output spectrum and pump laser spectrum based on pump modulation of various waveforms with and without phase difference are shown in Figure 5. The intensity difference of the laser output spectrum and the pump laser spectrum is due to the two acquisitions at different detection positions. According to the spectral data, we find that the influence of current waveform on the laser output spectrum is less. The existence of phase difference decreases the secondary absorption peak of the pump laser spectrum, which weakens the absorption of the secondary characteristic peak. This demonstrates pump modulation based on multiple current waveforms does not affect the laser output spectrum. For each current waveform, the impact of phase difference on the pump laser spectrum may be another possible reason to change the motion of MIP and RIG, thereby increasing the TMI threshold.

Table 1 summarizes the main experimental results, clearly showing that beam quality at the threshold is improved via pump modulation with multiple current waveforms. The pulse wave (duty cycle: 60%) can increase the TMI threshold to 245 W with a beam quality of 1.55. When the phase difference is modified, the average output power can continue to increase for each current waveform. With a modulation frequency of 2 kHz and phase difference of 440 μs, the TMI threshold reaches 270 W with a beam quality of 1.45. It can be inferred that pump modulation based on pulse wave (duty cycle: 60%) with certain phase differences is more conducive to energy transfer to the fundamental mode. In other experiments, when increasing the number of pump LDs with drivers from two groups to three groups, the maximum average output power of a stabilized beam is further increased to 380 W with pulse wave (duty cycle: 60%). This demonstrates that multiple modulation parameters can influence the energy transfer. It is interesting to note that multiple modulation parameters affect the pump suppression effect. Therefore, more attention needs to be paid to the pump modulation scheme for TMI mitigation both theoretically and experimentally.

## 4. Conclusions

In summary, we have demonstrated TMI mitigation via pump modulation based on multiple current waveforms in a fiber laser oscillator. Among sinusoidal wave, triangular wave, and pulse wave, the optimal waveform for TMI mitigation is pulse wave. The modulation frequency, amplitude, and duty cycle of pulse wave are the three main factors that determine the control effect of TMI. It is found that the phase difference between the signal channels of the function generator influence the TMI threshold for each waveform. The maximum average output power of a stabilized beam can reach 270 W under pulse wave (duty cycle: 60%) with a modulation frequency of 2 kHz and a phase difference of 440 μs. This increases the TMI threshold in CW mode by a factor of 1.5, and the beam quality is improved to 1.45. In addition, the stable output power can be further improved by increasing the number of groups of pump LDs with drivers.

## Figures and Tables

**Table 1:** The TMI threshold and its beam quality under CW and pump modulation based on different current waveforms.

Operation Mode	Frequency	Channel 1	Channel 2	Phase Difference	TMI Threshold	Beam Quality @ Threshold M^2^ (M^2^_x_, M^2^_y_)
Continuous Wave	/	/	/	/	180 W	1.81 (1.84 1.79)
Sinusoidal Wave	1.67 kHz	580–980 mV	560–960 mV	0°	195 W	1.65 (1.77 1.54)
Triangular Wave					227 W	1.63 (1.74 1.53)
Pulse Wave (Duty cycle: 50%)				0 μs	238 W	1.58 (1.61 1.56)
Pulse Wave (Duty cycle: 60%)				0 μs	245 W	1.55 (1.65 1.45)
Sinusoidal Wave	1.67 kHz	580–980 mV	560–960 mV	30.4°	221 W	1.55 (1.54 1.56)
Triangular Wave				30.3°	235 W	1.54 (1.53 1.55)
Pulse Wave (Duty cycle: 50%)				100 μs	247 W	1.53 (1.54 1.52)
Pulse Wave (Duty cycle: 60%)	2 kHz			440 μs	270 W	1.45 (1.47 1.44)

## Data Availability

The data that support the fundings of this study are available from the corresponding author upon reasonable request.

## References

[1] Zervas M.N., Codemard C.A. (2014). High power fiber lasers: A review. IEEE J. Sel. Top. Quantum Electron..

[2] Shi W., Fang Q., Zhu X., Norwood R.A., Peyghambarian N. (2014). Fiber lasers and their applications [invited]. Appl. Opt..

[3] Wang Y., Chen G., Li J. (2018). Development and prospect of high power doped fibers. High Power Laser Sci. Eng..

[4] Liu Z., Jin X., Su R., Ma P., Zhou P. (2019). Development status of high power fiber lasers and their coherent beam combination. Sci. China Inf. Sci..

[5] Jauregui C., Limpert J., Tünnermann A. (2013). High-power fibre lasers. Nat. Photonics.

[6] Zhang H., Zhou P., Xiao H., Leng J., Tao R., Wang X., Xu J., Xu X., Liu Z. (2018). Toward high-power nonlinear fiber amplifier. High Power Laser Sci. Eng..

[7] Stutzki F., Jansen F., Otto H.J., Jauregui C., Limpert J., Tünnermann A. (2014). Designing advanced very-large-mode-area fibers for power scaling of fiber-laser systems. Optica.

[8] Jauregui C., Stihler C., Limpert J. (2020). Transverse Mode Instability. Adv. Opt. Photonics.

[9] Eidam T., Hanf S., Seise E., Andersen T.V., Gabler T., Wirth C., Schreiber T., Limpert J., Tünnermann A. (2010). Femtosecond fiber CPA system emitting 830 W average output power. Opt. Lett..

[10] Eidam T., Wirth C., Jauregui C., Stutzki F., Jansen F., Otto H.J., Schmidt O., Schreiber T., Limpert J., Tünnermann A. (2011). Experimental observations of the threshold-like onset of mode instabilities in high power fiber amplifiers. Opt. Express.

[11] Beier F., Möller F., Sattler B., Nold J., Liem A., Hupel C., Kuhn S., Hein S., Haarlammert N., Schreiber T. (2018). Experimental investigations on the TMI thresholds oflow-NA Yb-doped single-mode fibers. Opt. Lett..

[12] Ward B., Robin C., Dajani I. (2012). Origin of thermal modal instabilities in large mode area fiber amplifiers. Opt. Express.

[13] Smith A.V., Smith J.J. (2016). Mode instability thresholds for Tm-doped fiber amplifiers pumped at 790 nm. Opt. Express.

[14] Smith A.V., Smith J.J. (2011). Mode instability in high power fiber amplifiers. Opt. Express.

[15] Hansen K.R., Alkeskjold T.T., Broeng J., Lægsgaard J. (2013). Theoretical analysis of mode instability in high-power fiber amplifiers. Opt. Express.

[16] Gaida C., Gebhardt M., Heuermann T., Wang Z., Stutzki F., Jauregui C., Limpert J. Observation of transverse-mode instabilities in a thulium-doped fiber amplifier. Proceedings of the Fiber Lasers XVI: Technology and Systems.

[17] Otto H.J., Stutzki F., Jansen F., Eidam T., Jauregui C., Limpert J., Tünnermann A. (2012). Temporal dynamics of mode instabilities in high power fiber lasers and amplifiers. Opt. Express.

[18] Jauregui C., Eidam T., Otto H.J., Stutzki F., Jansen F., Limpert J., Tünnermann A. (2012). Temperature-induced index gratings and their impact on mode instabilities in high-power fiber laser systems. Opt. Express.

[19] Stihler C., Jauregui C., Tünnermann A., Limpert J. (2018). Modal energy transfer by thermally induced refractive index gratings in Yb-doped fibers. Light Sci. Appl..

[20] Jauregui C., Eidam T., Otto H.J., Stutzki F., Jansen F., Limpert J., Tünnermann A. (2012). Physical origin of mode instabilities in high-power fiber laser systems. Opt. Express.

[21] Jauregui C., Stihler C., Tünnermann A., Limpert J. Transverse mode instabilities in burst operation of high-power fiber laser systems. Proceedings of the Fiber Lasers XV: Technology and Systems.

[22] Stihler C., Jauregui C., Tünnermann A., Limpert J. (2018). Phase-shift evolution of the thermally-induced refractive index grating in high-power fiber laser systems induced by pump-power variations. Opt. Express.

[23] Jauregui C., Stihler C., Tünnermann A., Limpert J. Origin and evolution of phase-shifts in high-power fiber laser systems: Detailed insights into TMI. Proceedings of the Fiber Lasers XVI: Technology and Systems.

[24] Stihler C., Jauregui C., Kholaif S.E., Limpert J. (2020). Intensity noise as a driver for transverse mode instability in fiber amplifiers. PhotoniX.

[25] Smith A.V., Smith J.J. (2012). Influence of pump and seed modulation on the mode instability thresholds of fiber amplifiers. Opt. Express.

[26] Stihler C., Jauregui C., Tünnermann A., Limpert J. The impact of pump power noise on transverse mode instabilities. Proceedings of the Fiber Lasers XVI: Technology and Systems.

[27] Jauregui C., Stihler C., Tünnermann A., Limpert J. (2018). Pump-modulation-induced beam stabilization in high-power fiber laser systems above the mode instability threshold. Opt. Express.

[28] Minden M. (2002). Phase Control Mechanism for Coherent Fiber Amplifier Arrays. U.S. Patent.

